# Dynamic Construction of Stimulus Values in the Ventromedial Prefrontal Cortex

**DOI:** 10.1371/journal.pone.0021074

**Published:** 2011-06-14

**Authors:** Alison Harris, Ralph Adolphs, Colin Camerer, Antonio Rangel

**Affiliations:** 1 Humanities and Social Sciences, California Institute of Technology, Pasadena, California, United States of America; 2 Computation and Neural Systems, California Institute of Technology, Pasadena, California, United States of America; 3 Biology, California Institute of Technology, Pasadena, California, United States of America; The University of Melbourne, Australia

## Abstract

Signals representing the value assigned to stimuli at the time of choice have been repeatedly observed in ventromedial prefrontal cortex (vmPFC). Yet it remains unknown how these value representations are computed from sensory and memory representations in more posterior brain regions. We used electroencephalography (EEG) while subjects evaluated appetitive and aversive food items to study how event-related responses modulated by stimulus value evolve over time. We found that value-related activity shifted from posterior to anterior, and from parietal to central to frontal sensors, across three major time windows after stimulus onset: 150–250 ms, 400–550 ms, and 700–800 ms. Exploratory localization of the EEG signal revealed a shifting network of activity moving from sensory and memory structures to areas associated with value coding, with stimulus value activity localized to vmPFC only from 400 ms onwards. Consistent with these results, functional connectivity analyses also showed a causal flow of information from temporal cortex to vmPFC. Thus, although value signals are present as early as 150 ms after stimulus onset, the value signals in vmPFC appear relatively late in the choice process, and seem to reflect the integration of incoming information from sensory and memory related regions.

## Introduction

A growing consensus in neuroscience suggests that simple choices are made by first assigning values to the stimuli under consideration and then comparing those values to select the best one [1]–[7]. Neural signals associated with the value assigned to stimuli at the time of choice have been reported both with electrophysiology [8], [9] and human neuroimaging [10]–[21]. Converging data from these techniques, as well as reports of impaired choice behavior in patients with focal brain lesions [22], suggest that explicit value representations in ventromedial prefrontal cortex (vmPFC) guide simple choices.

A critical open question is how stimulus value signals are computed in the vmPFC. One natural hypothesis is that the vmPFC receives multimodal information about stimulus attributes from more posterior sensory and association cortices, and then integrates this information into an overall stimulus value prior to choice [2]. This idea is motivated by previous studies showing that value signals in vmPFC can represent a variety of value attributes [9], [12], [13], [17], [19], together with the known network of connections between vmPFC and sensory [23]–[25] and limbic [26]–[28] cortices.

We used electroencephalography (EEG) together with a novel statistical analysis approach to quantify the dynamics of value-related neural activity while subjects evaluated appetitive and aversive food items. Based on the findings above, we hypothesized that stimulus value activity appears first in parietal and temporal areas, and that it emerges in the vmPFC relatively late in the valuation process.

We used EEG, rather than BOLD fMRI, because behavioral evidence shows that subjects can assign values to basic stimuli in less than 1000 ms [29], which makes the low temporal resolution of fMRI ill-suited to examine this problem. In contrast, the combination of EEG recordings with recent advances in signal processing and source reconstruction offers the ability to measure signals with high temporal resolution without excessive sacrifices in spatial localization.

## Methods

### Subjects

Twenty-three subjects (ages 18–40, 18 males) were recruited from the local Caltech community. All subjects were right-handed and had normal or corrected-to-normal vision. Six subjects were excluded from further analyses on the basis of predefined exclusion criteria: two exhibited restricted or unusual food preferences (vegetarian and/or dislike of snack foods), two reported confusion over task instructions during exit debriefing, and two exhibited EEG artifact (either the alpha wave was inseparable from evoked responses, or there were channels with persistent high variability and artifact (≥100 µV) after data cleaning). Subjects provided written consent prior to participation. All procedures were reviewed and approved by Caltech's Institutional Review Board (IRB).

### Stimuli

**Figure 1:**
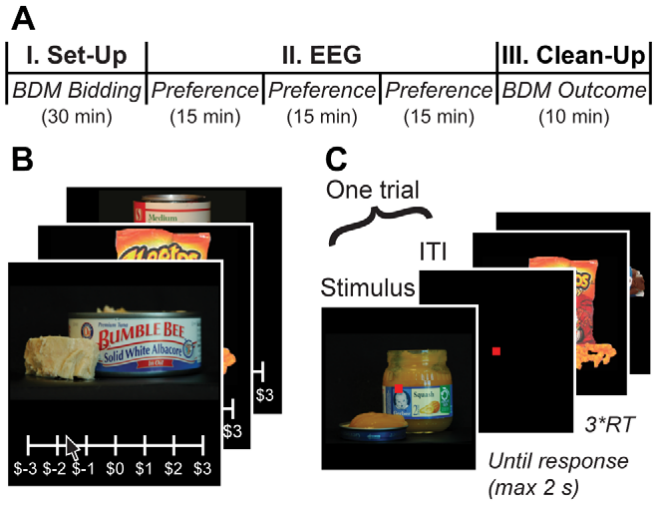
Experimental stimuli and procedure. (A) Experimental procedure. Part I: subjects placed self-paced bids for the opportunity to eat or avoid eating 60 different food items. Part II: subjects performed a liking-rating task for the same food items via a 4AFC button press (Strong Dislike, Weak Dislike, Weak Like, Strong Like) while their EEG responses were recorded. Part III: a randomly selected bidding trial was implemented that determined which food, if any, subjects had to eat. (B) Sample stimuli, bidding task screen. (C) Sample stimuli and liking-rating task screen (Part II).

Subjects were presented with color images of 60 food items (576×432 pixels, subtending 8.6°×6.8° of visual angle) presented on a black background (Figure 1). The specific food items were selected on the basis of prior behavioral data to span a wide range of desirability: from strongly disliked to strongly liked. Examples of appetitive items include snack potato chips and candy bars. Examples of aversive items include canned meats and baby foods.

### Task

Subjects did not eat for at least two hours prior to the experiment. The experiment consisted of three parts (Figure 1A).

First, subjects played a Becker-DeGroot-Marschak (BDM) bidding task [30]. In this part of the task subjects were given $$3 in cash and had to place bids for the opportunity to either eat or avoid having to eat the different food items at the end of the experiment. As part of the consent process, subjects agreed to eat whatever food item, if any, they received from the auction procedure at the end of the experiment. For each of 60 trials (one per food item), subjects were shown a food item and had to bid between $$-3 and $$3, with negative bid values indicating the amount of money that they demanded in order to eat a disliked item, and positive bid values indicating the amount that they were willing to pay to eat a liked item. A random bidding trial was selected at the end of the experiment and the outcome of that trial was implemented using the rules described below. Note that, as a result, subjects did not have to worry about spreading their $$3 budget over different items.

The rules of the BDM are as follows. Let *b* denote the subject's bid for a particular item. After the experiment, a random number *n* is drawn with equal probability from the distribution of $$-3 to $$3. If *n* is positive and *b*≥*n*, the subject pays *n* and receives the food item; if *b*<*n*, the subject does not receive the food item but does not pay. If *n* is negative, when *b*≤*n* the subject pays *n* and avoids eating the food item; otherwise, the subject does not pay but must eat the food item. This auction has the useful property that the approximate optimal strategy is to bid exactly one's willingness-to-pay for an item, since the actual price is determined by the random number *n* rather than the subject's bid. Thus, bids from this auction provide a good measure of the value (positive or negative) assigned to consuming each item. This approach has been used with success in several prior studies of reward processing [10], [14], [15].

Second, subjects participated in a liking-rating task. At this stage the EEG cap was positioned and adjusted as described below, and EEG activity was recorded while they indicated their preference for each of the same 60 items using a 4-point scale (Strong Dislike, Weak Dislike, Weak Like, Strong Like). This task consisted of 3 runs of 240 trials each, for a total of 720 trials. The 60 food items were presented randomly interleaved four times per run. Each item was shown for a maximum of 2 s and subjects had to enter a liking rating within this time frame. Subjects were asked to respond as quickly and accurately as possible before the image disappeared. They entered their responses using a keyboard, with two responses assigned to left middle and index fingers, and two assigned to right middle and index fingers (order counterbalanced across subjects). Subjects completed a short practice block of the liking-rating task before the actual EEG recordings began. Median RT was calculated cumulatively during each run, and the duration of the inter-trial interval (ITI) was iteratively adjusted to 3* median(RT). This procedure was used to make sure that all decision-making computations were completed before the start of the next trial. Given the relative length of stimulus presentation, and the concomitant difficulty in maintaining fixation, runs were subdivided into short blocks with intervening self-paced breaks. During the task subjects were asked to maintain central fixation and minimize eye movements and blinks, and their performance was monitored with the recording equipment. The three runs were separated by 10-minute breaks.

Third, the outcome of the BDM auction was implemented by the computer. In the last 10 minutes of the experiment, the experimenter paid the subject and observed the subject eating the food item, if necessary.

### EEG Data Acquisition and Pre-Processing

EEG data was collected using a 128-channel HydroCel Geodesic Sensor Net (Electrical Geodesics, Inc., Eugene, OR), with AgCl-plated electrodes in fitted sensor nets. Evoked brain potentials were digitized continuously at a sampling rate of 1000 Hz, filtered with 400 Hz low-pass and 0.1 Hz high-pass cut-off. Vertex electrode Cz served as reference during recording. Impedances for all channels were kept below 50 kΩ throughout the experiment, with adjustments during the 10-minute breaks. Although these re-adjustments could potentially affect signal quality across runs, slow voltage drifts associated with increased impedance minimally distort the averaged ERP and were cleaned from the data during artifact removal (see below).

Data pre-processing was performed offline using the EEGLAB toolbox [31] for MATLAB (Mathworks, Inc., Andover, MA). Following import, data were re-sampled to 500 Hz, re-referenced to an average reference, and notch filtered at 60 Hz. Epochs for each trial were extracted for a time window of 2300 ms (100 ms pre-, 2200 ms post), time-locked to the stimulus onset. The ERP data were sorted into conditions based on the subject's four possible liking ratings (Strong Dislike to Strong Like).

**Figure 2:**
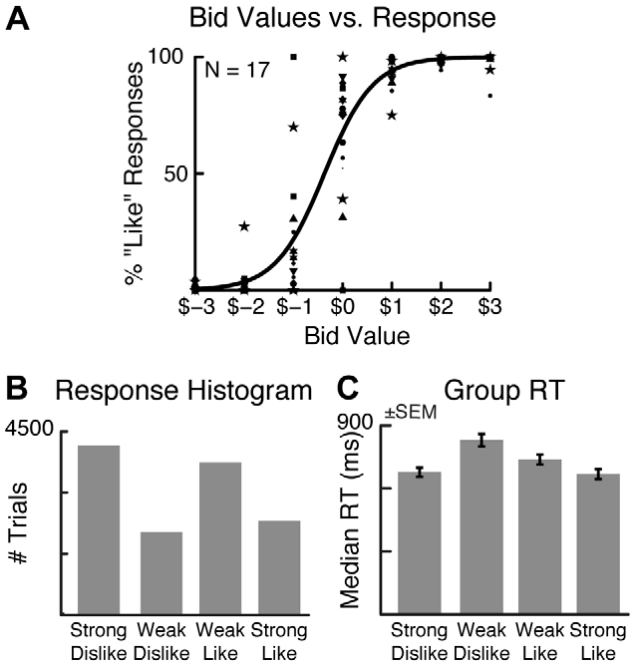
Behavioral data. (A) Comparison of bids and liking ratings. Although individual subjects (markers) varied in their utilization of the bidding range, there was a strong logistic relationship between bid values and preference ratings (thick line, average of individual fits). (B) Aggregate response histogram. (C) Average median RTs by liking rating.

Since subjects' ratings generally showed an asymmetrical distribution (Figure 2B), traditional artifact removal techniques based on trial rejection are suboptimal for this dataset. Given this, we identified and removed experimental artifacts using independent-components analysis (ICA), implemented via second-order blind identification (SOBI) [32], [33]. Like other blind source separation algorithms, SOBI enables the effective identification of artifactual components, which can be removed while leaving the overall number of trials in each condition intact [33]. Specifically, SOBI linearly “unmixes” the EEG data into a sum of temporally correlated and spatially fixed components, which can be classified as artifactual or non-artifactual based on their power spectra, scalp topographies, and activity. Task-related non-artifactual components are characterized by clear stimulus- or response-locking and meaningful scalp topography, whereas artifactual components reflect blinks, eye movement, muscle activity, sensor noise, and slow voltage drifts. By projecting only non-artifactual components back onto the scalp, it is possible to obtain artifact-corrected brain signals [34]. This approach is therefore unlikely to distort the temporal structure of the data to the same extent as conventional FIR filtering [35].

**Figure 3:**
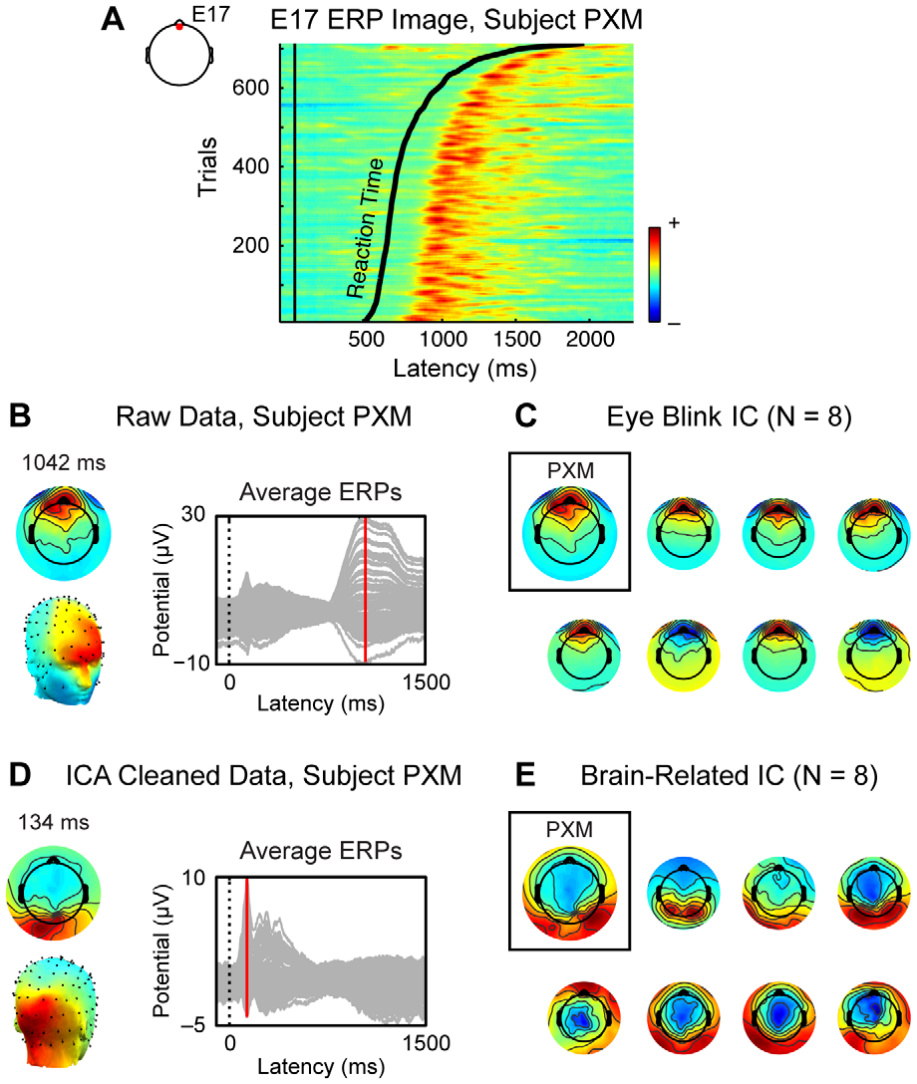
Artifact removal via independent component analysis (ICA). (A) Trial-by-trial activity from a frontal sensor (E17, left) in a subject (PXM) with excessive blinking, sorted by reaction time. This subject's pattern of blinking after the response creates large positive deflections at this sensor following key press (black line). (B) Topographic scalp representations at 1042 ms (left) show a stereotyped frontal pattern consistent with eye blink, visible as a large deflection in the ERP (right). (C) ICA analysis extracts a component corresponding to the eye blinks, as indicated here by the scalp topography. Such components are also visible in the data of other subjects with less obvious artifacts in their EEG data (7 subjects shown here). The polarity of the independent component is arbitrary. (D) PXM's data following ICA cleaning. In contrast to the raw data, the major peaks now reflect brain-related activity. (E) Corresponding brain-related ICA components in PXM and the other depicted subjects.

Given our interest in source reconstruction, artifacts related to eye movements and blinks present a particular concern, as they may be incorrectly projected onto the ventral prefrontal region of interest. These movements produce highly stereotyped patterns of activity at electrodes near the eyes: for example, eye blink is characterized by a large deflection in sensors immediately above the eyes (Figure 3B, left). Likewise, horizontal saccades are associated with lateralized activity in sensors near the orbits [36]. Although eye movements and blinks were not directly measured in our experiment, we were able to identify and remove patterns consistent with these artifacts using ICA (Figure 3). Trial-by-trial data from a frontal sensor (Figure 3A) in one subject (PXM) shows clear deflections during the period after response (black line), visible as a large deflection in the average evoked response (Figure 3B, right). In this subject and others, ICA isolated a component with the stereotyped scalp distribution of eye blink (Figure 3C), which also showed trial-by-trial activity consistent with blinks. Removal of this and other artifactual components produced a cleaned data set isolating brain-related activity (Figure 3D–E).

From this cleaned data, we then constructed the datasets used in our analyses. First, we constructed a stimulus-locked data set of epochs of 1100 ms (100 ms pre-, 1000 ms post-) that were baseline-corrected to the pre-stimulus period, and sorted into experimental conditions based on each subject's liking ratings. We also created an alternative response-locked dataset that was conducted on the same data by extracting 600 ms epochs (−400 to 200 ms) aligned to the response time. To avoid any confounds related to average decision-making computations, this data set was also baseline-corrected to the 100 ms pre-stimulus period prior to the extraction of response-locked epochs.

### Mixed Effects Linear Regression Analysis

To characterize the time course of value signal computations, each subject's data were separately entered into a linear regression analysis. Evoked data for each trial between 100 and 1000 ms post-stimulus-onset were integrated over 50-ms windows for each channel. The EEG responses for each of the 128 sensors×18 time windows were then entered into a linear regression model of the form
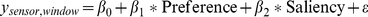
where the dependent variable y_sensor,window_ consists of the trial-by-trial data (in µV) for a particular sensor and time window, and β_0_ is the average activity in the sensor. The Preference covariate measures the underlying value of the item, as measured by the liking rating, which was coded from 1 ( = Strongly disliked) to 4 ( = Strongly liked). The Saliency covariate measures the strength of preference, regardless of valence, and it is coded as 1 ( = Weakly liked or disliked) and 2 ( = Strongly liked or disliked). (Note that “saliency” in this context is analogous to arousal, rather than perceptual saliency.) We included the saliency covariate to take out the components of the signal that are related to saliency (as in [14]), which increases the statistical power of the linear model to identify value-related activity. The response-locked data were modeled in a similar fashion from −400 to 200 ms, but with smaller windows of 40 ms due to the shorter epoch length.

In each case, the linear regression analysis produced a map of estimated regression coefficients for every sensor, time window, and subject. We aggregated these maps into mixed-effect group estimates by computing one-sample t-tests versus zero across subjects for each sensor and time window. Given that the preference and saliency regressors were not fully orthogonal for allsubjects, we also estimated a version of this model in which the preference regressors were orthogonalized to the saliency regressors.

Given the large number of separate tests (128 channels * 18 time windows), this analysis presents a significant multiple comparisons problem. Because the false discovery rate (FDR) is often overly conservative in multichannel ERP data [37], we corrected for multiple comparisons using permutation tests [37], [38]. This was implemented by re-running the linear models for every possible permutation of the 4 condition labels (4! = 24). The t-statistics from each permuted regression were then sorted in ascending order, and the observed values from the actual data compared to this distribution of values. Values greater than or equal to the highest t values, and less than or equal to the lowest t values, were considered as surviving multiple comparisons correction with a threshold of *p* = 0.04 (one-tailed). Given the small number of permutations possible, in practice this meant that in order to survive correction the observed data had to fall at the extreme end of the permutation distribution.

Finally, we also estimated an additional linear mixed-effects model to compare the coding of value in the appetitive and aversive categories. Similar to above, the EEG data were entered into a linear regression with a constant term, a predictor for saliency, and predictors for preference modulated by positive valence (0 = Disliked, 3 = Weak Like, 4 = Strong Like) and negative valence (0 = Liked, 1 = Strong Dislike, 2 = Weak Dislike). Thus, the modulators indicated both magnitude and valence for positive and negative preference.

### 3D Source Reconstruction

We performed exploratory distributed source reconstruction of the EEG signals using SPM8 (Wellcome Department of Imaging Neuroscience, Institute of Neurology, London, UK). To look for source activity that modulated the EEG signal by preference, we computed the contrast waveform in each subject using a linear contrast of the form [−3 −1 +1 +3], which weights the data to find monotonic increases in response with increasing preference. (These values are arbitrary, with the only restrictions being hat they sum to zero and correspond to equal, monotonically increasing steps between levels. Note that this construction is commonly used to test for linear trends.) The individual waveforms were then aggregated using a group inversion source reconstruction algorithm that models hundreds of small patches on the cortical sheet as potential sources [39]. Whereas single subject reconstruction optimizes both the priors and estimated sources, group inversion applies the additional constraint that the same set of sources must explain responses in all subjects [39], [40]. Group analysis then simply entails passing the source reconstructions from individual subjects to a second-level ANOVA model, from which statistical parametric maps (SPM) of significant group activity can be obtained.

A key precondition for such group analyses is that all subjects' reconstructed activity must be within the same source space. In our data, this was ensured via use of a “canonical mesh” based on SPM's template head model, derived from the MNI brain. Sensors were coregistered with the MRI coordinate system using a generic template of EGI sensors and fiducials provided with the SPM software. Additionally, the remaining sensor locations were matched to the cortical mesh using an iterative alignment algorithm. The source space was modeled using a boundary element model, in which the different tissues of the head (e.g., cerebrospinal fluid, skull, skin) are approximated by closed triangle meshes with different conductivity values [41].

The output of the source reconstruction for each subject consisted of predicted time courses of source activity in all potential sources across the entire epoch length (e.g., −100 to 1000 ms for stimulus-locked data). We could then test for significant sources of activity across subjects by computing an F test on the level of activity in each source at each time window of interest. Note that because source reconstructions were performed on the parametric difference waveforms, and the polarity of evoked potentials is not necessarily meaningful, the contrast of interest was an F statistic. Significant sources in each time window of interest were visualized in terms of maximal intensity projection (MIP) of the F statistic, thresholded with family-wise-error (FWE) correction at *p*<0.05. FWE-corrected F values for each cluster of source activity in the stimulus-locked analysis, and corresponding MNI coordinates, are displayed in Tables S1, S2, S3.

To validate this analysis, we also performed localizations for evoked responses with known cortical sources, including the visual evoked potential (VEP) and movement-related cortical potential. The procedure was largely similar, but used waveforms averaged across all conditions (VEP), or by motor response hand. Statistics were calculated for time windows of 100 to 150 ms after stimulus onset and −100 to 0 ms pre-response, respectively, using an uncorrected threshold of *p*<0.00005.

We can also employ the source reconstruction data as a “virtual electrode.” This allows us to read out the activity associated with each source over the time course of the trial. For select regions of interest (ROIs), the time course of each maximal intensity projection (MIP) was computed within each subject by using forward modeling as implemented in SPM8, with the time course for each XYZ coordinate of the ROI calculated separately and the resulting responses averaged.

### Causal Connectivity Analysis

We performed a functional connectivity analysis of the EEG signals identified by the 3D source reconstruction using Granger causality [42], [43]. This methodology compares two simultaneously measured time-varying signals X and Y, and considers signal X to be “causal” if past information about X improves prediction of signal Y.

In the three time windows identified by the linear regression, we focused on a small subset of the sources localized by the distributed reconstruction: (1) 150–250 ms, left lingual gyrus (LG) and right superior temporal gyrus (STG); (2) 400–550 ms, bilateral vmPFC and left BA46; and, (3) 700–800 ms, right intraparietal sulcus (IPS). These areas were specifically chosen due to their high statistical significance in the source localization, known connectivity with vmPFC, and proposed role in sensory processing or stimulus valuation.

For each source, a region of interest (ROI) was defined from the second-order group analysis using dipole clusters surviving the FWE-corrected threshold of *p*<0.05 (see Tables S1, S2, S3 for cluster-level and local maxima). For each subject and ROI cluster, a MIP time course was computed for each dipole in the cluster, and these were then averaged to produce subject-level ROI activations used in connectivity analysis. Although some researchers have used individual dipoles instead of overall ROI activation to avoid averaging-related temporal smoothing [44], preliminary examination of our data showed no advantage for individual dipole activations, perhaps reflecting our use of an averaged difference waveform for source reconstruction.

Granger causality analysis was performed using the Granger Causal Connectivity Analysis toolbox (GCCA) [43] for Matlab. In each subject, MIP activations for each ROI were extracted in 3 time windows that immediately followed the preference-related responses in the ERP data: 250–400 ms, 550–700 ms, and 800–950 ms after stimulus onset. The goal was then to investigate how activity recorded during the windows associated with the preference responses (e.g., 150–250 ms) affected Granger causal connectivity in the subsequent window (i.e., 250–400 ms). Preprocessing consisted of linear detrending, subtraction of the temporal mean, and division by the temporal standard deviation. The data across subjects were then combined into a single matrix, in which each subject was treated as one “realization” of a single underlying stochastic process, followed by removal of the ensemble mean and division by the ensemble standard deviation.

A major assumption of Granger causality is covariance stationarity (CS): i.e., that the mean and variance of the time series do not change over time. To address the covariance non-stationarity commonly present in EEG signals, we applied first-order differencing to the data. We then performed two common tests of covariance stationarity on the first-order differenced data: the Augmented Dickey-Fuller (ADF) and KPSS tests. The ADF test failed to reject the null hypothesis of the presence of a unit root, and thus it cannot rule out the presence of non-stationarities. In contrast, the KPSS test, which has a null hypothesis of no unit root, did not reject the null hypothesis, implying no unit root. These divergent results provide no clear evidence regarding covariance stationarity, and additional differencing necessary for convergence (5–6 iterations) would lead to interpretative difficulty. Thus, the first-order differencing procedure was considered sufficient to approximate the stationarity assumptions required by the Granger connectivity analysis.

For each time window, the optimal model order was selected using the Bayesian information criterion (BIC), corresponding to 6 (250–400 and 550–700 ms) or 7 (800–950 ms), a lag of 12–14 ms. Significance was assessed using a threshold of *p* = 0.01, Bonferroni-corrected. Validity was verified using the Durbin-Watson test, which found no significant correlation of the residuals, and the consistency test, which showed high consistency of the fitted model with the correlation structure of the data (>95%).

## Results

### Behavioral Data

We verified that subjects' preference ratings were consistent with decision values during actual choices by comparing the percentage of “Like” responses (both Strong and Weak) for each food item to the bid values collected prior to recording. Although subjects differed in their use of the full scale of possible bid values (Figure 2A), their preference data was well-described by a logistic regression: a one-sample t-test on the sensitivity of like responses on bid value was highly significant (t(16) = 9.2, *p* = 9×10^−8^). Thus, the preference ratings were consistent with the decision values assigned to foods at the time of choice.

The indifference point for the fitted logistic function showed a slight negative bias (mean indifference point = $$−0.4). Though small, this negative shift was highly significant across subjects (t(16) = −4.3, *p* = 6×10^−4^), suggesting that subjects were generally more willing to pay to avoid foods they considered aversive than to obtain foods they considered appetitive. However, since our comparison of interest is the relative ordering of preferences rather than their absolute magnitudes, this bias is unlikely to affect the EEG analysis.

Comparison of the preference rating assigned to each food item across subjects revealed a basic continuum from aversive to appetitive, though with high inter-subject variability (Figure S1). Given this high variability in preference rating, low-level image features specific to the different food categories are unlikely to account for differences in EEG response. In contrast to the high variability between subjects, within-subject preference ratings for each item were stable across the three experimental runs (*F*<1).

The preference ratings were divided among the four response categories, with a larger proportion of “Strong Dislike” and “Weak Like” responses (Figure 2B). Median reaction times were significantly faster for the strong, compared to weak, preference conditions (Figure 2C), as indicated by a repeated-measures ANOVA (*F*(3,48) = 42.2, *p* = 2×10^−13^). This inverted U-shaped function of median RT by condition was confirmed by a contrast analysis testing for a quadratic trend, which revealed a significant effect (t(16) = −10.1, *p* = 2×10^−8^).

### Stimulus-Locked Analysis of the EEG Data

The first step of the analysis was to look for sensors at which recorded activity correlated with the preference ratings at different time windows, which enabled us to trace the dynamics of value signals as they appear in different parts of the brain. The analysis was conducted by estimating a mixed-effects linear regression for each sensor and time window. For each subject, the data between 100 and 1000 ms post-stimulus-onset were integrated over 50-ms bins to produce 18 time windows of activity for each sensor and trial. The evoked activity (in µV) in each of the 128 sensors×18 time windows was fed into a linear regression in which preference was the main regressor of interest. The estimated coefficients for each subject, sensor, and window were aggregated into mixed-effect estimates by computing one-sample t-tests versus the null hypothesis that the coefficient is zero in that sensor and time window; all p-values were corrected for multiple comparisons using permutation tests.

**Figure 4:**
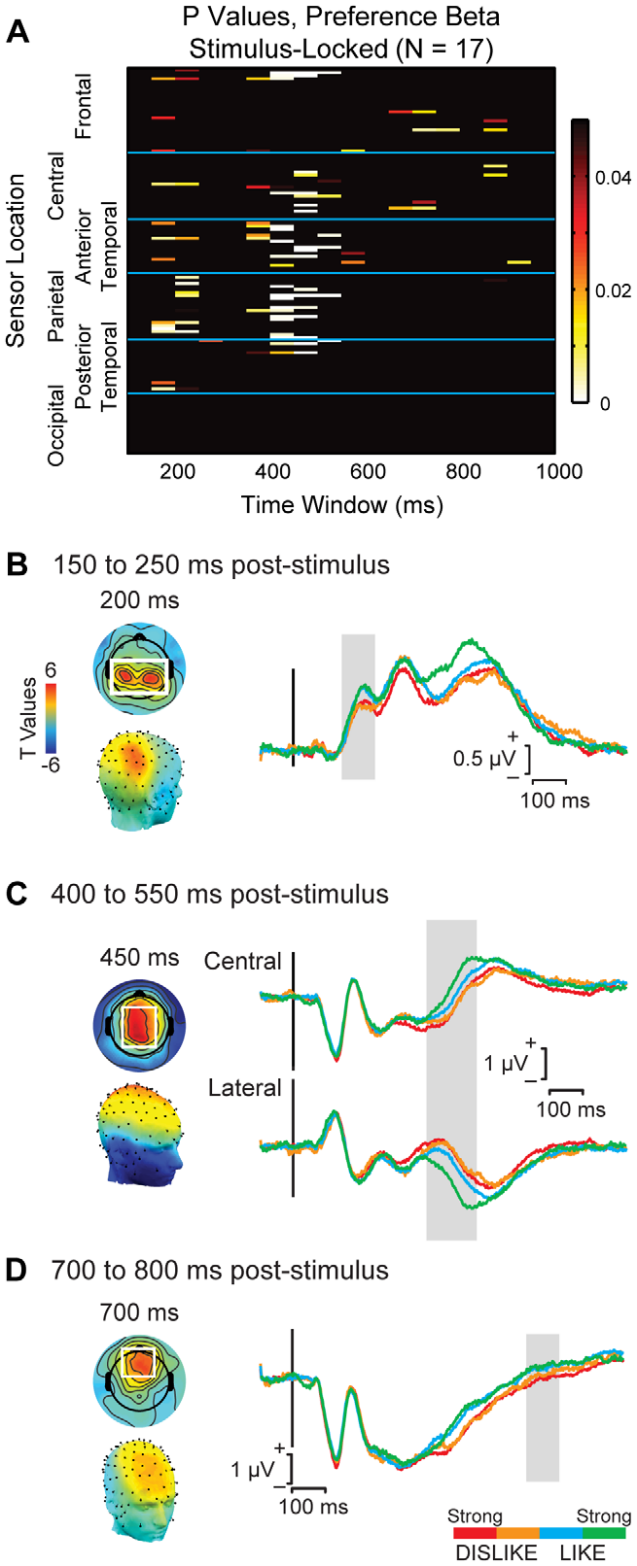
Value signals in the stimulus-locked data. (A) Heat map summarizing the results of the linear regression analysis in the 128 sensors and 18 time windows (each 50 ms long). Each box indicates the p-value for a mixed-effects group estimate of the effect of liking rating on the activity of a specific sensor during a specific time window. P-values are corrected for multiple comparisons using a permutation test. (B) ERP responses in parietal sensors during the 150 to 250 ms time window. (C) ERP responses in central and frontotemporal sensors during the 400 to 550 ms time window. (D) ERP responses in frontal sensors during the 700 to 800 ms time window. In each case, the left maps show the scalp distributions of t-values for the liking rating variable in the linear regression in two- (top) and three-dimensional (bottom) projections. The sensors of interest (SOIs) from which the ERP is extracted are shown in the white boxes. ERPs were computed by averaging waveforms first within and then across subjects. In each case the ERP shows a parametric response that correlates with the liking ratings during the time window highlighted in gray. In the 400–550 ms window, the SOIs also included bilateral frontal and lateral sensors with high negative t values.

Three key patterns emerged from the analysis of preference-related activity (Figure 4A). First, an initial wave of widely distributed responses appeared around 150–250 ms, with the most significant responses taking place in parietal sensors. This was followed by another wave of responses around 400–500 ms that included central, frontal, and anterior temporal sensors. Third, around 700–800 ms there is restricted but significant activity in the frontal sensors.

We characterized these patterns in more detail by identifying sensors of interest (SOIs) in each time window, defined as sensors in which activity was significantly modulated at *p*<0.01 by preference. Figure 4B–D depict key SOIs for the different time windows discussed above, as well as grand average waveforms for the sensors, separated by condition. Note that in each case there is clear separation of activity by response category during the time window of interest.

Given the unbalanced distribution of preference ratings in most subjects, we also estimated a version of the model with orthogonalized regressors (see methods for details). This additional analysis generated similar results: a t-test comparing the estimated betas in the original and orthogonalized-regressors models (z transformed to account for scaling) found no significant differences between them (all *p*s>0.9). Therefore, all further analyses focused on the original model.

The results for the saliency regressors are described and discussed in Materials S1 and Figure S2. We also estimated an additional regression model to compare the coding of valence for appetitive and aversive items. Although this model has less statistical power, the results suggest a similar pattern of activity (Figure S3).

These results suggest a shifting pattern of preference-related activity moving from posterior to anterior cortices over time, in line with recent results on the coding of high- versus low-energetic images of food [45]. However, since this analysis was carried out at the sensor level, it lacked anatomical specificity about which particular regions were responsible for the observed pattern. We performed an exploratory examination of this question using a distributed source localization technique.

### Distributed Source Reconstruction: Stimulus-Locked Analysis

To explore the possible neural sources underlying the evoked response, we used a Bayesian distributed source reconstruction analysis. A useful feature of the analysis is that it allowed us to localize the sources of the signal of interest in different time windows, one individual at a time, and then aggregate the individual activations to construct statistical parametric mappings of the different sources at the group level.

Note that, given the uncertainty surrounding available reconstruction techniques, these results are exploratory and must be interpreted with caution. As described in the Methods, care must be taken to remove ocular artifacts, which otherwise may be incorrectly projected onto the ventral prefrontal region of interest. Additionally, individual variability in head shape, brain structure, and cortical folding can translate to large spatial uncertainty in source localization. This is particularly true for our data, as individual differences in head shape, sensor position, and brain anatomy were not considered in our analysis. Instead, the sensor data for all subjects were realigned into a canonical neuroanatomical space, limiting the spatial resolution of the source reconstruction. These results should therefore be considered rough estimates of the underlying sources, with unknown spatial error.

**Figure 5:**
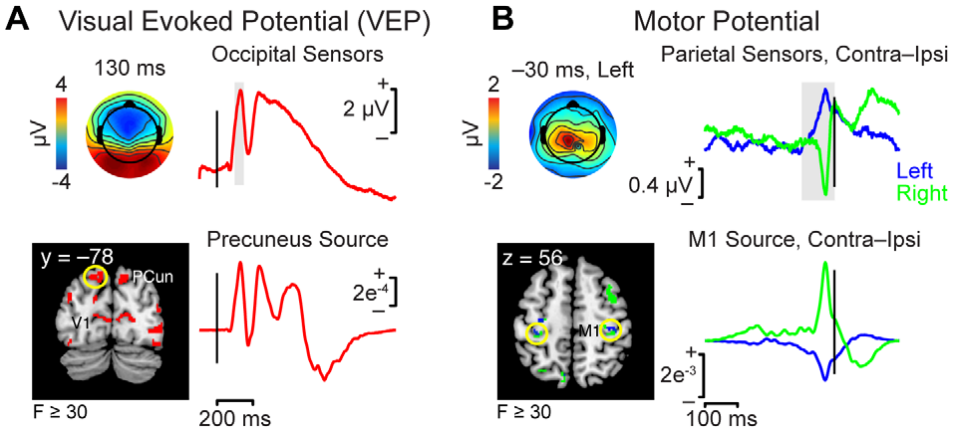
Validation of the distributed source reconstruction technique. Note that the spatial resolution of reconstruction is limited due to inherent constraints and the realignment of subjects into a common neuroanatomical space. Depicted spatial precision reflects the high statistical threshold used: F≥30, corresponding to *p*<0.00005 (uncorrected). (A) Visual evoked potential (VEP). Top: Topography and waveform of the VEP recorded from occipital sensors. The time window used for source reconstruction (100–150 ms) is shown in gray. Bottom: Sources of the VEP included visual areas such as primary visual cortex (V1), middle and inferior occipital gyrus, and precuneus (PCun). Activity from one maximal intensity projection (MIP) (circled) is highly similar to the original EEG data. (B) Movement-related cortical motor potential. Top: Topography and waveform of the motor potential recorded from parietal sensors, for the comparison of contralateral versus ipsilateral motor output. Bottom: Time courses of sources localized to M1 (circled) show a similar contra-ipsi pattern.

Bearing in mind the exploratory nature of this technique, we validated the source reconstruction methodology using two ERP signals with well-known anatomical loci: the visual evoked potential (VEP) and the movement-related cortical motor potential. Figure 5 displays the ERP components and corresponding sources with an F value of 30 or more, corresponding to a statistical threshold of approximately *p*<0.00005, uncorrected. (Note that this high statistical threshold accounts for the apparent spatial precision of the sources in this and the following figures.)

Peaking 80 to 130 ms after stimulus onset (Figure 5A, top), the earliest components of the VEP show response patterns consistent with low-level visual processing, including sensitivity to visual field location and image properties such as contrast [46]. Co-localization of these signals with fMRI activation has found sources in multiple visual areas, including striate and extrastriate cortex [47]. Consistent with these results, our exploratory source reconstruction showed sources throughout visual cortex, including V1, inferior and middle occipital gyrus, and precuneus (Figure 5A, bottom left). Using the source reconstruction data as a “virtual electrode” to read out the modeled neural activity for one such region (circled), the time course of the maximal intensity projection (MIP) shows a pattern similar to the activity recorded across occipital sensors (Figure 5A, right).

Although a number of movement-related cortical potentials have been identified in the time leading up to response [48]–[50], we focused on the late period approximately −100 ms prior to key press. The onset of motor response in this time window is commonly associated with a focal peak in movement-related activity (Figure 5B, top), visible in recordings from subdural electrodes over the corresponding hand motor representation [51]. Our preliminary source localization within this window likewise revealed activity in regions including primary motor cortex (M1, circled), for which MIP time courses showed the same contra >ipsi pattern seen in the original data (Figure 5B, bottom).

**Figure 6:**
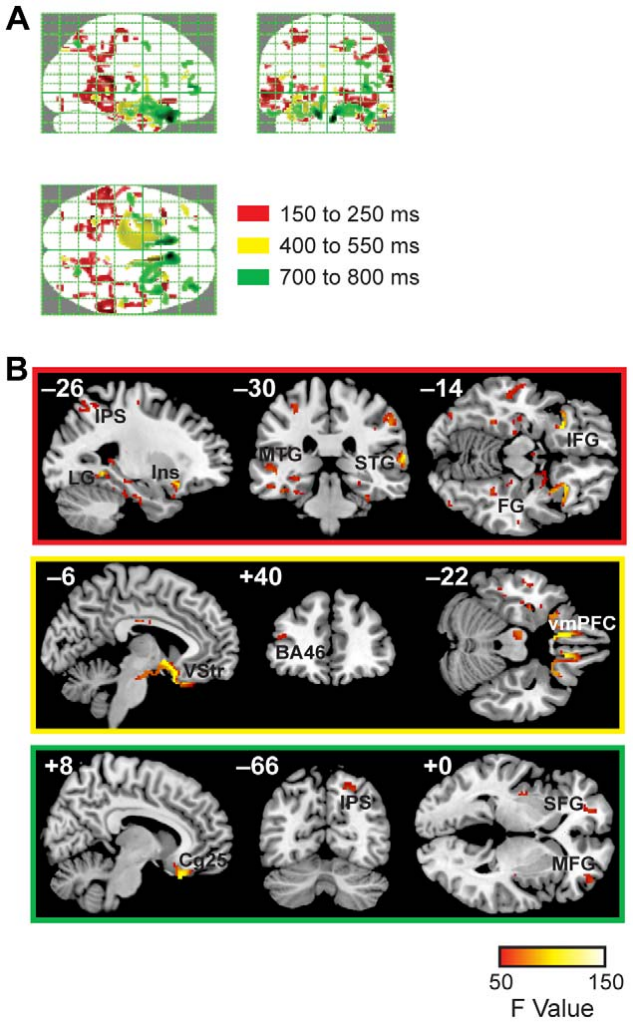
Exploratory distributed source reconstruction, stimulus-locked data. Depicted spatial precision reflects the high statistical threshold used: *p*<0.05, FWE-corrected. (A) Glass brain displays showing the maximum intensity projections (MIP) of all sources for each time window. (B) Representative sources of interest for each time window superimposed on the MNI brain. Top: 150 to 250 ms. Prominent sources include: fusiform and lingual gyrus (FG, LG), middle and superior temporal gyrus (MTG, STG), and parahippocampal gyrus; insula (Ins), associated with gustatory processing; and inferior frontal gyrus (IFG). Middle: 400 to 450 ms. While posterior sources are still visible, sources associated with value computation emerge within this time window, including ventral striatum (VStr), Brodmann area 46 (BA46), and ventromedial prefrontal cortex (vmPFC). Bottom: 700 to 800 ms. In this time window, overlapping with average median RT (710 ms), there is continued activity in subgenual cingulate (Cg25) and intraparietal sulcus (IPS), as well as emerging activity in middle and superior frontal gyrus (MFG, SFG). See also Tables S1, S2, S3.

Thus, for established evoked potentials, exploratory source reconstruction localizes activity to areas consistent with known anatomical sources. Proceeding with source localization of our data, inference was carried out at a FWE-corrected threshold of *p*<0.05 and extent cluster size of 5 voxels. The same threshold was used to generate all figures. Again, these results must be interpreted with care given the coarse spatial resolution of the reconstruction technique. Nonetheless, the sources surviving our strict statistical threshold (Figure 6, Tables S1, S2, S3) are highly consistent with previous neuroimaging results [10]–[21].

In the earliest time window, from 150 to 250 ms, activity was localized primarily to bilateral “clusters” in temporal and parietal regions. Consistent with the scalp distribution of activity, these included a number of regions of parietal cortex, such as intraparietal sulcus and precuneus, as well as structures in the occipital, inferotemporal, and medial temporal lobes (see Table S1 for a complete list). These early sources are interesting because these areas have been widely associated with attention, memory, and object recognition based on the visual stream. In addition, sources of interest were also identified in inferior frontal gyrus (IFG) and anterior insula.

In the middle time window, 400–550 ms, activity continued to be localized to areas of the temporal lobe, with increasing activity in ventromedial prefrontal cortex, subgenual cingulate, and ventral striatum, structures known to be associated with coding of stimulus value (Table S2). Intriguingly, during this period a source was also visible in a region of dorsolateral prefrontal cortex overlapping with Brodmann area 46, the activity of which has previously been shown to correlate with goal values [10], [11] and to be implicated in the choice process [12], [13].

Finally, during the 700–800 ms window, sources were visible primarily in the vmPFC and anterior frontal cortex, with some activity in insula and intraparietal sulcus (Table S3). Given that this period coincides with the median observed reaction time, these sources may reflect a shift to response preparation and output.

### Causal Connectivity Analysis

Both of the previous analyses suggest a dynamic pattern for the computation of the value signal that begins in the temporal lobe and spreads over time to the vmPFC. We carried out a more formal test of this pattern using Granger causal connectivity analysis [43] on the neural activity projected from sources identified in the distributed reconstruction.

As a large number of dipole clusters survived the statistical threshold for exploratory source localization (Tables S1, S2, S3), to include all sources would have produced a model of extreme complexity that would be difficult to interpret. Therefore, we chose to focus instead on a small subset of sources with known roles in sensory processing or stimulus valuation and high statistical significance in the distributed reconstruction. Given the coarse spatial resolution of the localization, these regions of interest (ROIs) include a large number of dipoles from adjacent brain areas, and should not be treated as precise anatomical regions. Rather, these sources represent broad cortical divisions for which connectivity analysis can provide an exploratory measure of causal interrelationship.

In particular, we looked at the three time windows suggested by the linear regression analysis, selecting regions from the source reconstruction associated respectively with sensory processing, stimulus valuation, and motor planning. In the early window (150–250 ms), one chosen cluster (L LG) contained dipoles in lingual, fusiform, and parahippocampal cortex, regions known to be involved in visual object recognition [52]. Another ROI (R STG) spanned sections of right superior temporal sulcus, angular gyrus, and supramarginal gyrus, associated with visuospatial awareness and multimodal sensory convergence [53]. During the 400–550 ms window, we selected sources implicated in decision-making: left and right vmPFC and BA46. Finally, for the late 700–800 ms time window, intraparietal sulcus (R IPS) was chosen for its role in transforming goal values into motor output [54]. We then extracted activity measures from these same areas for the subsequent time windows: 250–400 ms, 550–700 ms, and 800–950 ms. Because this last period falls after the median average RT, in-depth analysis focused on the first two time windows. ROIs were defined from dipole clusters in the group analysis surviving an FWE-corrected threshold of *p*<0.05 (see Tables S1, S2, S3). Average MIP time courses were extracted from these ROIs in each subject, and the resulting matrix of MIP activations by sources was entered into a causal connectivity analysis. The basic idea of the Granger causal connectivity analysis is to test if activity in each area for a given time window ‘predicts’ activity in other areas in the follow-up time period.

**Figure 7:**
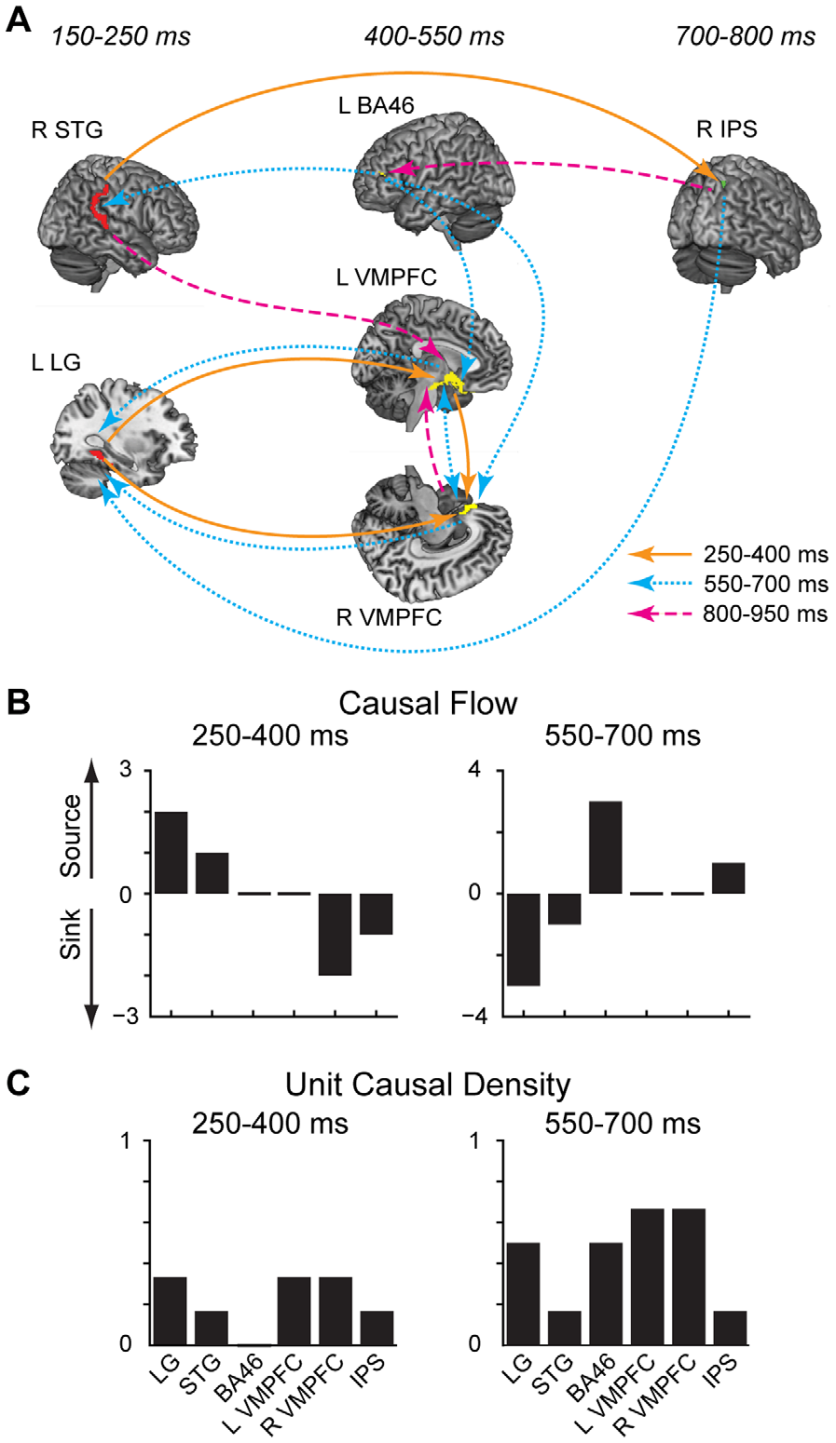
Preliminary Granger causal connectivity analysis. Note that due to the coarse spatial resolution of the source reconstruction technique, the selected regions of interest (ROIs) are not precise anatomical regions but rather represent broad divisions of cortex. (A) Model and causal connectivity across 3 time windows of interest (orange: 250–500 ms, cyan dotted: 550–700 ms, magenta dashed: 800–950 ms). All displayed connections are significant for a threshold of *p* = 0.01, Bonferroni-corrected. (B) Causal flow measures of the difference between the number of outgoing and incoming causal connections. Large positive values indicate “causal sources,” nodes with strong causal influence on the system, while negative values signify “causal sinks.” (C) Unit causal density measures of the summed causal interactions for a given node. High unit causal density indicates the presence of a hub in the causal network.

Figure 7A displays all connections for which Granger causality was significant at *p* = 0.01, Bonferroni-corrected, across all three time windows of interest. Following the earliest preference-related activity, 250 to 400 ms (orange lines), there are connections to bilateral vmPFC from a left temporal ROI encompassing lingual, parahippocampal, and fusiform gyrus (L LG), as well as within right parietal areas. The middle 550–700 ms period (dotted cyan lines) is characterized by recurrent inputs from vmPFC to temporal cortex, and the emergence of BA46 as a causal source to vmPFC and right temporoparietal regions (STG, supramarginal, and angular gyrus). Finally, in the late window following response, temporoparietal and parietal regions play a causal role in activity in BA46 and vmPFC. Also notable is the continued interplay between left and right vmPFC across the time course of response.

The causal dynamics of these time windows were formally quantified via measures of causal flow (Figure 7B) and unit causal density (Figure 7C). Causal flow, defined as the difference of in-flow versus out-flow, gives a sense of the causal influence exerted on the system by each node. For example, from 250–400 ms, temporal and temporoparietal regions are major causal sources; whereas, right vmPFC is a causal sink, receiving inputs from left vmPFC and LG. Likewise, in the later 550–700 ms period, BA46 shows a strong positive causal flow, reflecting its increased activity as a causal source. Unit causal density for a given node consists of its summed causal interactions, and can be used to identify causal hubs. In the 550–700 ms period, the vmPFC ROIs show the greatest unit causal density, reflecting their many connections.

These results support the idea that early value-related activity encoded in the temporal lobe is passed over time to vmPFC, with the information computed in vmPFC then feeding back to posterior regions. Additionally, the model shows a causal role for BA46 connections with vmPFC and temporoparietal regions.

### Response-Locked Analysis of EEG Data

Given that the source reconstruction shows increasing activity in vmPFC leading up to the time of response, one natural question is how the ERP responses vary with reaction time. We first analyzed this question by splitting the stimulus-locked data by median latency, which revealed similar preference-related activity for fast and slow RT trials (see discussion in Materials S1 and Figure S4).

A drawback of this approach is that it blurs response signals due to the variation in RTs across subjects and trials. To address this limitation we repeated the previous analysis using response-locked data with a 400-ms time window preceding response.

**Figure 8:**
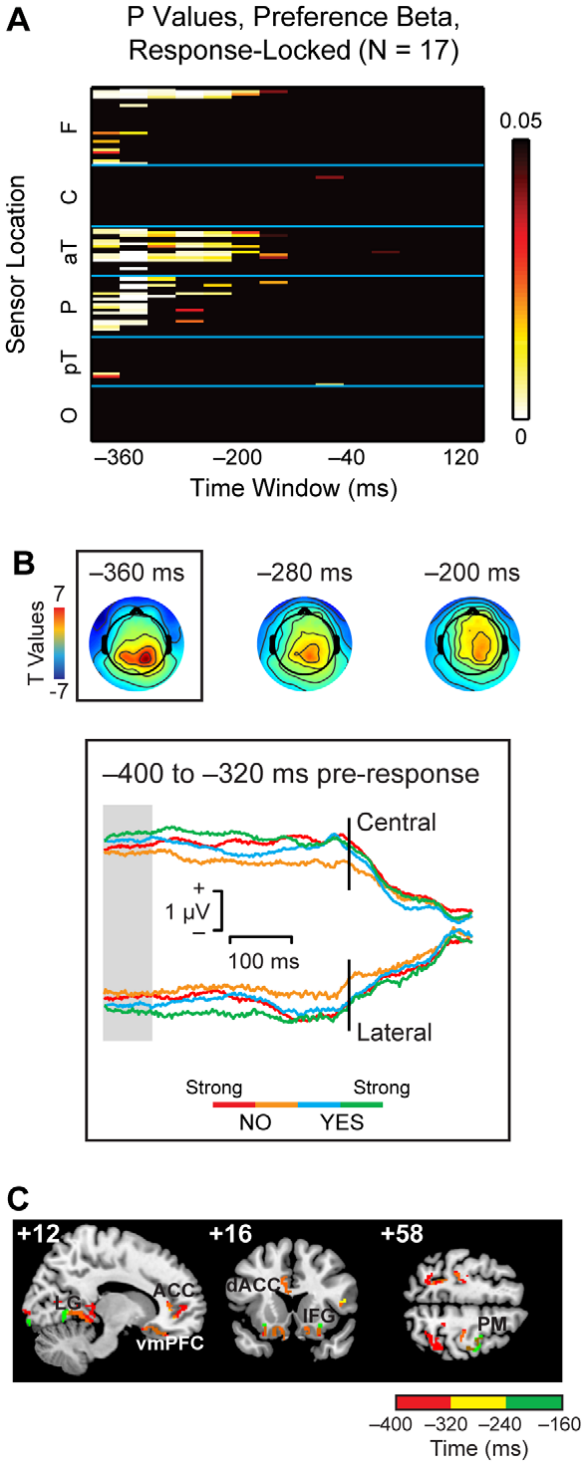
Value signals in the response-locked data. (A) Heat map summarizing the results of the linear regression analysis in the 128 sensors and 14 40-ms time windows. Each box indicates the p-value for a mixed-effects group estimate of the effect of liking rating on the activity of a specific sensor during a specific time window. P-values are corrected for multiple comparisons using a permutation test. (B) Top: Scalp distribution of t-values for the liking rating variable over time. Bottom: ERP grand average waveforms for preference, −400 to −320 ms pre-response. (Because significant effects of preference were largely restricted to the same electrodes over time, only this time window is displayed as an example.) The ERP shows a parametric response that correlates with the liking ratings during the time window highlighted in gray. (C) Exploratory distributed source reconstruction for the response-locked data. Identified sources were similar to those in the stimulus-locked analysis. Activity was also localized to anterior and dorsal cingulate cortex (ACC, dACC) and premotor cortex (PM), regions known to be engaged in response selection and planning.

Figure 8A displays the results of the mixed-effects regression analysis. Significant linear effects are visible from roughly −400 to −160 ms prior to response. Consistent with the stimulus-locked data, responses in this period were largely distributed across parietal, anterior temporal, and frontal sensors. Scalp maps at selected time points show a comparable topographic progression from parietal electrodes to more central sensors, with sustained preference-related activity at anterior temporal and frontal sensors (Figure 8B, top). Grand average waveforms, displayed for a sample window from −400 to −320 ms pre-response (Figure 8B, bottom), also show ordering by preference rating.

Similarly, signal localization over 80-ms time windows produced roughly similar sources to those seen in the stimulus-locked analysis, including areas of the lingual gyrus, superior temporal gyrus, insular and inferior frontal cortex, and vmPFC (Figure 8C). These data again support the idea that vmPFC responses reflect the integration of information about stimulus attributes coming from sensory and mnemonic structures, particularly in the temporal and parietal lobes. Reconstruction of the response-locked data also found a number of additional sources associated with selection and planning of the motor response, including regions of anterior, dorsal, and posterior cingulate cortex (−400 to −240 ms), and premotor cortex (−240 to −160 ms).

### EEG Sources versus fMRI Localization of Value Signals

**Figure 9:**
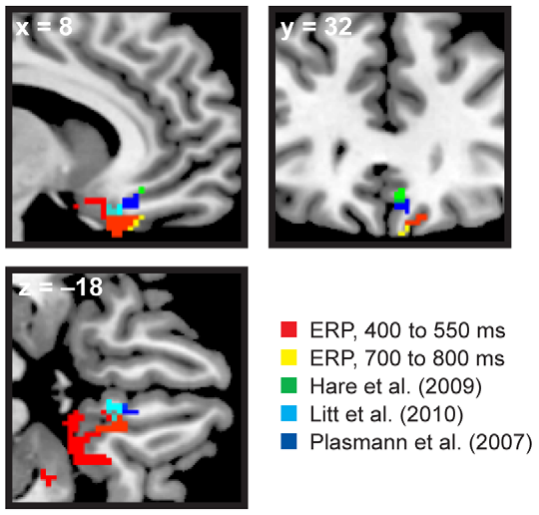
Stimulus-locked source reconstruction maps (FWE-corrected *p*<0.01) versus mOFC activations in 3 published fMRI studies. The source reconstruction of the EEG data from 400–550 ms (red) and 700–800 ms (yellow) shows vmPFC sources consistent with previous results from fMRI (green, cyan, and blue). Notably, the closest match with the EEG data comes from Litt et al. (2010), who used a similar evaluation task with appetitive and aversive stimuli.

Separate source reconstructions of stimulus- and response-locked data consistently localized value signals to the vmPFC. How do these sources relate to the regions of interest defined in fMRI? Although the spatial precision of EEG source reconstruction is too coarse to definitively address this question, a direct comparison of the EEG sources to published fMRI data shows an intriguingly similar locus of activity (Figure 9). The figure was constructed by taking spherical masks (4 mm radius) around the peak locus of activation in mOFC from three previous fMRI studies of valuation ([12], green; [14], cyan; [10], blue). Masks of right vmPFC from 400 to 550 ms and 700 to 800 ms with FWE-corrected *p*<0.01 are displayed in red and yellow. (In all cases, only the right hemisphere loci are shown for clarity.)

The vmPFC sources from EEG are in relatively close proximity to the more spatially-precise fMRI data, suggesting activation of roughly the same valuation areas in all of these experiments. Interestingly, the nearest fMRI region of interest to the EEG sources is that identified in Litt et al. (2010), which used a nearly analogous task in which subjects evaluated appetitive and aversive foods.

## Discussion

A basic open question in decision neuroscience is how the value signals that have been widely observed in vmPFC are actually computed. We found ERP responses significantly associated with value over three major time windows: 150–250 ms, 400–550 ms, and 700–800 ms after stimulus onset. Across these three epochs, the distribution of value-related activity shifted from posterior to anterior, and from parietal to central to frontal sensors. Consistent with this pattern, exploratory source reconstruction analyses localized activity to vmPFC only from 400 ms onwards, with a Granger connectivity analysis revealing significant causal connections between temporal and vmPFC sources. Source reconstruction of response-locked data was largely consistent with the stimulus-locked localization, additionally finding activity in cingulate and premotor cortex in the time leading up to response.

Together, these data identify a putative network of brain regions for dynamic value computation, encompassing posterior cortices and medial temporal lobe structures. Activity from 150–250 ms after stimulus onset was localized to parietal and temporal lobe structures, including fusiform, lingual, and parahippocampal gyri, hippocampus, and intraparietal sulcus, as well as the inferior frontal gyrus and insula. Although source reconstructions must be interpreted with caution, given the individual variability in head shape and brain anatomy, these sources match the known anatomical connections of vmPFC [24], [55], and also constitute plausible substrates for conveying sensory and mnemonic associations into the value signal computation.

These results place a lower bound on the time course of value signal computation, with preference-related activity as early as ∼150 ms after stimulus onset. By separating the linear ordering of preference from effects of saliency (Figure S2), this analysis demonstrated that ERP components associated with early sensory processing encode value information rather than stimulus saliency alone.

Compared to responses in posterior cortices, localization of evoked responses in vmPFC occurred relatively late, around 400 ms after stimulus onset. Again, these results are exploratory and should be interpreted with care, given the abovementioned spatial uncertainty in the reconstruction, as well as the potential mislocalization of eye movement artifacts. Yet this time course is consistent with the idea of integration across stimulus attributes, which might be fed to the vmPFC over hundreds of milliseconds as complex stimuli are perceived and associated semantic information is retrieved from memory.

However, this long latency appears at odds with single-unit recordings, which have reported value signals in vmPFC across the time course of choice [8], and as early as 100–150 ms after stimulus onset [56]. These discrepancies may reflect experimental, technical, and functional issues. Experimentally, the studies of Padoa-Schioppa and Assad used highly learned stimuli that were depicted using simple geometrical color symbols. These features of the experimental design are likely to have reduced the need for stimulus attribute decoding and integration. Furthermore, EEG represents the synchronous activity of tens of thousands of neurons, whereas early value signals have been reflected in decreased firing rates of single neurons with already low spontaneous rates [56], which would be difficult to detect in the evoked response. Likewise, value coding that peaked within 500 ms of onset was reported only for a few hundred neurons out of a sample of nearly a thousand [8], again making it unlikely that such responses would be detected in the aggregate EEG signal. Therefore, while single neurons may encode value as soon as 150 milliseconds following stimulus presentation, our data suggest that robust responses across a large neural population occur relatively later.

Such an interpretation is also in line with findings from the domain of visual perception, where it has been proposed that OFC activity facilitates object recognition [57]. In this view, value may be assigned to sensory attributes through multiple cycles of feedback between prefrontal cortex and more posterior sensory and mnemonic structures. Although we failed to observe early value signals in vmPFC, perhaps due to the technical and theoretical issues stated above, the later large response could reflect the culmination of this iterative process, in which diverse sensory attributes are bound into a representation for value computation.

Our results are consistent with the known strong connectivity of vmPFC to sensory areas [24], [25], [55], which put it in a privileged position for integrating attribute information. Anatomically positioned close to olfactory cortex and gustatory regions of the insula [25], vmPFC also features connections to visual inferotemporal cortex [24], as well as memory-related structures such as the amygdala, hippocampus, and entorhinal and perirhinal cortex [26]–[28]. Combined with links to cognitive control regions such as ACC [58] and DLPFC [23], [59] these interconnections are suggestive of the role of vmPFC as a value attribute integrator. Supporting this idea, recent work using a binary probabilistic categorization task in fMRI suggests that vmPFC integrates perceptual evidence encoded in ventral temporal cortex [60]. Further experiments currently being conducted in our laboratory should further help to distinguish the temporal hallmarks of stimulus valuation from those associated with choice.

Together, these data illustrate shifting networks of cortical activity over the time course of stimulus valuation. The dynamics of stimulus valuation across the brain are marked by a progression from representation of sensory attributes to more abstract conceptual codes for stimulus value.

## Supporting Information

Materials S1 The supporting materials provide details of supplementary analyses of saliency effects, the coding of appetitive versus aversive value, and the variation of stimulus-locked ERP responses with reaction time.
(DOC)

Figure S1 Inter-subject variability in food ratings. Foods are sorted by mean rating on a 4-point scale from 1 (lowest) to 4 (highest). The median rating for each item is indicated by the central mark, with the edges of the box delineating the 25^th^ and 75^th^ percentiles. Although there is a basic continuum from aversive to appetitive, ratings varied tremendously from subject to subject.
(TIF)

Figure S2 Saliency signals in the stimulus-locked data. (A) Heat map summarizing p-values for the mixed-effects group estimate of the effect of saliency (Strong vs. Weak), corrected for multiple comparisons using a permutation test. (B) Scalp distributions of saliency-related activity, 250–450 ms. (C) ERP responses in posterior (left) and anterior (right) sensors during the 3 time windows associated with saliency effects. Note that the response from 450–500 ms appears to reflect an asymmetric response to the Strong Like condition, rather than a true saliency effect.
(TIF)

Figure S3 Coding of appetitive versus aversive value. (A) Scalp topographies at 450 ms for the original analysis of linear preference coding (left) versus positive (middle) and negative (right) valence. (B) Heat maps summarizing the p-values of betas from the mixed-effects regression on valence, for positive (top) and negative (bottom) valence. Note that these analyses are not corrected for multiple comparisons.
(TIF)

Figure S4 Split-latency analysis. For each condition and subject, the median RT was used to divide trials into fast (<median) and slow (>median) RTs. We then estimated the linear model separately for both types of trials. (A) Corrected mixed-effects p-value map for the sensors and time windows exhibiting activity in fast- (top) and slow-RT (bottom) trials. (B) Corrected p-value map for the paired t-test comparison of preference modulation in slow- versus fast-RT trials. (C) Average waveform data for a single sensor of interest (left, in green) that was chosen based on having significant parametric responses for both the 400–550 ms time window in fast-RT data, and the 700–800 ms window for slow-RT data.
(TIF)

Table S1 Peak MNI coordinates, source reconstruction 150–250 ms. Clusters surviving FWE-corrected threshold *p*<0.05 (*F* = 55.5) and cluster size threshold *k* = 5. In this and all other tables of peak coordinates, bold indicates a cluster-level maximum, with separate (>8 mm) maxima listed in plain type below. Because of the relatively low spatial resolution of EEG reconstruction, the source regions listed here do not precisely correspond to the coordinates, but rather reflect the general location of source activity within the specified cluster. *Denotes clusters used as regions of interest (ROIs) in causal connectivity analysis.
(DOC)

Table S2 Peak MNI coordinates, source reconstruction 400–550 ms. Clusters surviving FWE-corrected threshold *p*<0.05 (*F* = 56.5) and cluster size threshold *k* = 5. * Denotes clusters used as ROIs in causal connectivity analysis.
(DOC)

Table S3 Peak MNI coordinates, source reconstruction 700–800 ms. Clusters surviving FWE-corrected threshold *p*<0.05 (*F* = 55.9) and cluster size threshold *k* = 5. * Denotes clusters used as ROIs in causal connectivity analysis.
(DOC)
