# Supplementary material for: Dynamic Construction of Stimulus Values in the Ventromedial Prefrontal Cortex
Source: PLoS One. 2011 Jun 14;6(6):e21074. doi: 10.1371/journal.pone.0021074 (PMC3114863; doi:10.1371/journal.pone.0021074)
Supplement: Materials S1 — The supporting materials provide details of supplementary analyses of saliency effects, the coding of appetitive versus aversive value, and the variation of stimulus-locked ERP responses with reaction time. (DOC) [file pone.0021074.s001.doc]

**Supplementary Materials**

*Saliency Effects*

In addition to the main analysis of preference, we also examined the beta values for saliency (Strongly liked/disliked vs. Weakly liked/disliked). Unlike preference-related activity, saliency signals were heterogeneous in terms of their scalp distributions and time course (Figure S2A), with no clear posterior-to-anterior movement over time (Figure S2B). Instead, a subset of both anterior and posterior sensors were significant for a number of time windows, including 250-300, 350-400, and 450-500 ms after stimulus onset. However, grand average waveforms suggest that only the first two of these time periods reflect true saliency effects, with the significant result from 450-500 ms driven by sensors with an asymmetrical response to Strong Like versus other conditions (Figure S2C). Thus the bulk of saliency effects in our data occur between 250 and 400 ms after stimulus onset; compared to preference-related activity, these effects are of relatively short duration and heterogeneous scalp distribution.

Source reconstruction of saliency effects in the 250 to 400 ms time window (using contrast [+1 –1 –1 +1]) failed to find significant dipoles for FWE-corrected *p* = 0.05.

*Coding of Appetitive vs. Aversive Value*

Although this study focuses on parametric preference-related ERP responses, another common distinction in the value computation literature is that of appetitive versus aversive stimuli or events. Neural dissociations between appetitive and aversive coding have been reported for several distinct types of value signals, including those associated with expected value, actual value, and prediction error (see for a review). Therefore, one obvious question is whether there is separable coding for the liked and disliked conditions, or whether they reflect extremes of a single value dimension.

To examine this issue, we performed an additional linear regression with separate predictors for positive and negative valence modulated by preference rating. Results are presented in Figure S3; note that due to the lower power of this analysis, results are not corrected for multiple comparisons. Scalp maps of the original parametric preference beta and the valence terms at 450 ms show highly similar topographies, though the negative valence is distributed slightly more posteriorly (Figure S3A). Comparison of significance by sensor and time window likewise shows highly similar patterns of activity in the positive and negative valence conditions. Particularly in the 400-550 ms time window, where a t-test found no significant difference between positive and negative valence, this distribution of activity is also strikingly similar to the original parametric analysis of preference (Figure 4A).

Thus, both appetitive and aversive stimuli appear to be represented along a single value dimension, rather than via separable neural codes. While we did not perform source reconstruction for these comparisons due to the low power of this analysis, the scalp topographies of positive and negative valence in the 400-550 ms range are similar to preference-related activity that has been localized to vmPFC (Figure 6). This idea is consistent with recent results from fMRI finding representations of both appetitive and aversive stimuli within medial OFC .

*Split-Latency Analysis of EEG Data*

We examined how the ERP responses vary with reaction time by re-running the linear regression analysis in two sub-samples of the stimulus-locked EEG data, split by median latency (computed separately for each subject): a fast RT sample (trials with RT < median RT) and a slow RT sample (trials with RT > median RT).

Mixed-effects linear regression analyses for fast and slow RTs found the same set of sensors exhibiting modulation by preference in the 150-250 ms and 400-550 ms time windows, with activity differing significantly only in the later 700-800 ms range (Figure S4A). Consistent with the idea that slower RTs are associated with extended or additional value computations, activity in the frontal and central sensors was significantly stronger in the slow sample around the 700-800 ms window (Figure S4B). Likewise, grand average waveforms for a sensor with significant preference-related activity in both the fast- and slow-RT datasets show different time courses for preference-related responses, with a later effect for slow RTs (Figure S4C).

Reference:

1. Plassmann H, O'Doherty JP, Rangel A (2010) Appetitive and aversive goal values are encoded in the medial orbitofrontal cortex at the time of decision making. J Neurosci 30: 10799-10808.
