# Supplementary figures and images for: Dynamic Construction of Stimulus Values in the Ventromedial Prefrontal Cortex
Source: PLoS One. 2011 Jun 14;6(6):e21074. doi: 10.1371/journal.pone.0021074 (PMC3114863; doi:10.1371/journal.pone.0021074)

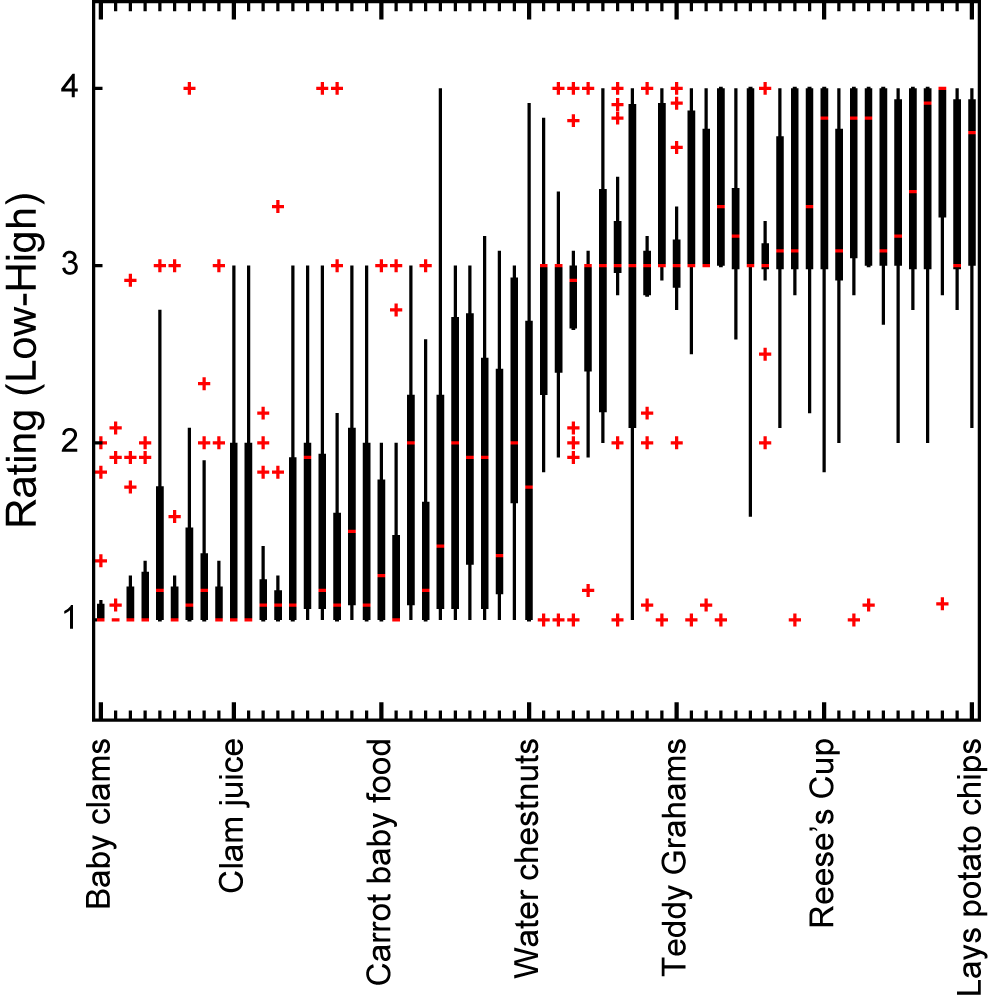

Supplement: Figure S1 — Inter-subject variability in food ratings. Foods are sorted by mean rating on a 4-point scale from 1 (lowest) to 4 (highest). The median rating for each item is indicated by the central mark, with the edges of the box delineating the 25th and 75th percentiles. Although there is a basic continuum from aversive to appetitive, ratings varied tremendously from subject to subject. (TIF) [file pone.0021074.s002.tif]

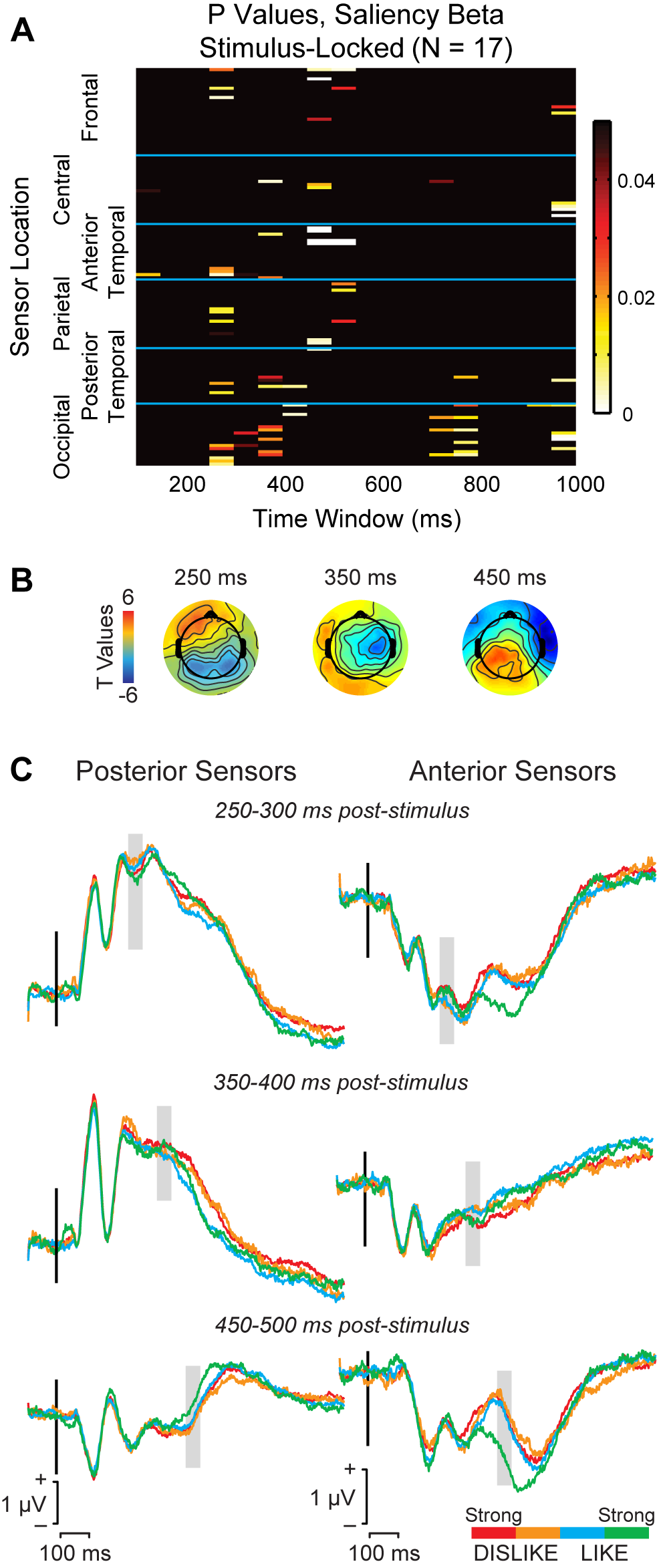

Supplement: Figure S2 — Saliency signals in the stimulus-locked data. (A) Heat map summarizing p-values for the mixed-effects group estimate of the effect of saliency (Strong vs. Weak), corrected for multiple comparisons using a permutation test. (B) Scalp distributions of saliency-related activity, 250–450 ms. (C) ERP responses in posterior (left) and anterior (right) sensors during the 3 time windows associated with saliency effects. Note that the response from 450–500 ms appears to reflect an asymmetric response to the Strong Like condition, rather than a true saliency effect. (TIF) [file pone.0021074.s003.tif]

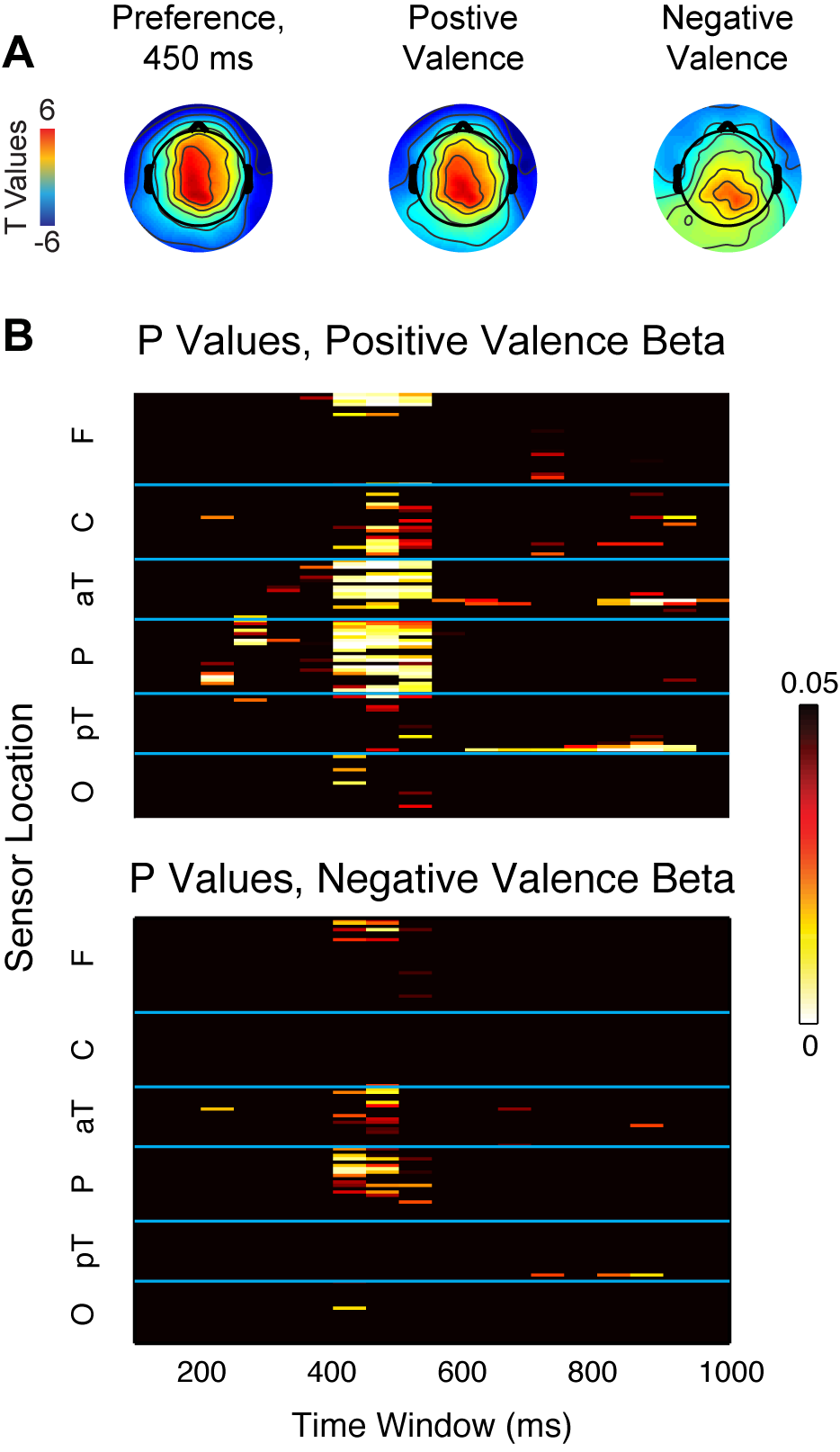

Supplement: Figure S3 — Coding of appetitive versus aversive value. (A) Scalp topographies at 450 ms for the original analysis of linear preference coding (left) versus positive (middle) and negative (right) valence. (B) Heat maps summarizing the p-values of betas from the mixed-effects regression on valence, for positive (top) and negative (bottom) valence. Note that these analyses are not corrected for multiple comparisons. (TIF) [file pone.0021074.s004.tif]

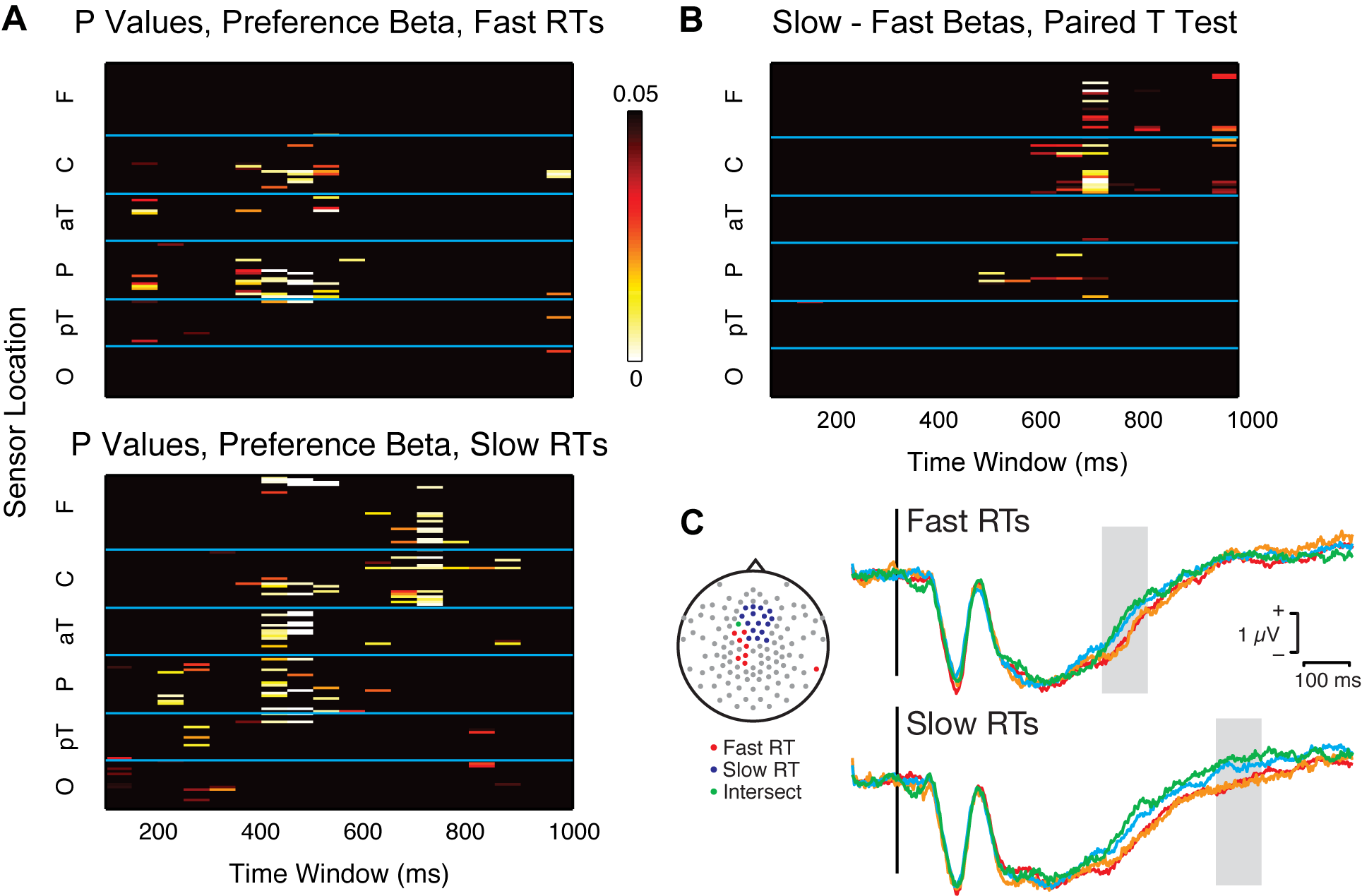

Supplement: Figure S4 — Split-latency analysis. For each condition and subject, the median RT was used to divide trials into fast (<median) and slow (>median) RTs. We then estimated the linear model separately for both types of trials. (A) Corrected mixed-effects p-value map for the sensors and time windows exhibiting activity in fast- (top) and slow-RT (bottom) trials. (B) Corrected p-value map for the paired t-test comparison of preference modulation in slow- versus fast-RT trials. (C) Average waveform data for a single sensor of interest (left, in green) that was chosen based on having significant parametric responses for both the 400–550 ms time window in fast-RT data, and the 700–800 ms window for slow-RT data. (TIF) [file pone.0021074.s005.tif]
