# Supplementary material for: Dynamic Construction of Stimulus Values in the Ventromedial Prefrontal Cortex
Source: PLoS One. 2011 Jun 14;6(6):e21074. doi: 10.1371/journal.pone.0021074 (PMC3114863; doi:10.1371/journal.pone.0021074)
Supplement: Table S1 — Peak MNI coordinates, source reconstruction 150–250 ms. Clusters surviving FWE-corrected threshold p<0.05 (F = 55.5) and cluster size threshold k = 5. In this and all other tables of peak coordinates, bold indicates a cluster-level maximum, with separate (>8 mm) maxima listed in plain type below. Because of the relatively low spatial resolution of EEG reconstruction, the source regions listed here do not precisely correspond to the coordinates, but rather reflect the general location of source activity within the specified cluster. *Denotes clusters used as regions of interest (ROIs) in causal connectivity analysis. (DOC) [file pone.0021074.s006.doc]

**Table S1.** Peak MNI Coordinates, Source Reconstruction 150-250 ms.

| *# Voxels* | *Side* | *Peak MNI Coordinates* | | | *F* | *MNI Coordinate Region* |
| --- | --- | --- | --- | --- | --- | --- |
| 146 | L | **–32** | **–48** | **–6** | **169.8** | Lingual gyrus* |
|  |  | –30 | –34 | –22 | 75.7 | Parahippocampal gyrus |
|  |  | –14 | –52 | –2 | 69.4 | Fusiform gyrus |
| 78 | L | **–26** | **20** | **–14** | **141.2** | Inferior frontal gyrus |
|  |  | –36 | 20 | –14 | 97.1 | Inferior orbitofrontal gyrus |
|  |  | –32 | 2 | –16 | 57.9 | Insula |
| 203 | R | **64** | **–32** | **12** | **119.4** | Superior temporal gyrus* |
|  |  | 64 | –42 | 18 | 109.8 | Supramarginal gyrus |
|  |  | 54 | –30 | 42 | 87.0 | Inferior parietal lobule |
| 146 | R | **26** | **20** | **–12** | **114.8** | Inferior frontal gyrus |
|  |  | 32 | 10 | –14 | 97.8 | Inferior orbitofrontal gyrus |
|  |  | 34 | 2 | –18 | 82.8 | Insula |
| 271 | L | **–62** | **–34** | **–2** | **104.2** | Middle temporal gyrus |
|  |  | –50 | –50 | 10 | 79.8 | Superior temporal gyrus |
|  |  | –62 | –42 | –8 | 75.8 |  |
| 142 | R | **20** | **–54** | **0** | **99.5** | Lingual gyrus |
|  |  | 30 | –32 | –26 | 83.2 | Parahippocampal gyrus |
|  |  | 18 | –56 | –10 | 75.7 | Fusiform gyrus |
| 58 | R | **42** | **14** | **–30** | **80.8** | Anterior temporal |
| 16 | L | **–36** | **–80** | **–16** | **80.5** | Occipital lobe |
| 24 | L | **–32** | **–16** | **16** | **80.4** | Insula |
|  |  | –54 | –10 | 12 | 70.2 |  |
|  |  | –44 | –12 | 16 | 66.7 |  |
| 17 | R | **34** | **–46** | **36** | **79.9** | Parietal lobe |
| 57 | L | **–28** | **–16** | **58** | **78.2** | Precentral gyrus |
|  |  | –32 | –26 | 62 | 69.5 |  |
|  |  | –32 | –32 | 50 | 62.3 |  |
| 78 | R | **58** | **–16** | **–18** | **74.6** | Middle temporal gyrus |
|  |  | 44 | 0 | –32 | 71.1 |  |
|  |  | 58 | 2 | –24 | 69.6 |  |
| 11 | L | **–42** | **–30** | **–20** | **74.3** | Temporal lobe |
| 9 | R | **18** | **–76** | **46** | **73.7** | Precuneus |
|  |  | 8 | –78 | 46 | 59.0 |  |
| 31 | R | **24** | **–64** | **50** | **72.9** | Intraparietal sulcus |
| 45 | L | **–22** | **–66** | **60** | **71.4** | Superior parietal lobule |
| 61 | L | **–20** | **–16** | **–28** | **68.8** | Parahippocampal gyrus |
|  |  | –30 | –14 | –26 | 68.2 | Medial temporal lobe |
|  |  | –22 | –26 | –26 | 55.9 |  |
| 42 | L | **–52** | **–34** | **–16** | **68.3** | Middle temporal gyrus |
|  |  | –64 | –16 | –12 |  |  |
| 5 | L | **–40** | **–40** | **50** | **67.3** | Inferior parietal lobule |
| 13 | L | **–58** | **–8** | **–24** | **67.0** | Middle temporal gyrus |
| 5 | L | **–34** | **52** | **6** | **66.5** | Middle frontal gyrus |
| 6 | R | **36** | **–8** | **56** | **64.8** | Frontal lobe |
| 8 | L | **–58** | **–38** | **28** | **64.6** | Inferior parietal lobe |
| 19 | R | **48** | **–64** | **–2** | **64.3** | Middle temporal gyrus |
| 20 | R | **38** | **–82** | **–8** | **64.2** | Inferior occipital gyrus |
| 31 | R | **12** | **–50** | **46** | **63.6** | Precuneus |
|  |  | 10 | –60 | 44 | 63.4 |  |
| 8 | R | **48** | **–46** | **–6** | **63.4** | Middle temporal gyrus |
| 55 | L | **–30** | **–30** | **–8** | **60.6** | Hippocampus |
|  |  | –24 | –24 | –12 | 59.0 | Posterior cingulate |
|  |  | –10 | –48 | 6 | 58.3 |  |
| 8 | R | **30** | **–36** | **–10** | **58.2** | Parahippocampal gyrus |
| 5 | R | **16** | **–34** | **–10** | **58.1** | Lingual gyrus |

Clusters surviving FWE-corrected threshold *p* < 0.05 (*F* = 55.5) and cluster size threshold *k* = 5. In this and all other tables of peak coordinates, bold indicates a cluster-level maximum, with separate (>8 mm) maxima listed in plain type below. Because of the relatively low spatial resolution of EEG reconstruction, the source regions listed here do not precisely correspond to the coordinates, but rather reflect the general location of source activity within the specified cluster. *Denotes clusters used as regions of interest (ROIs) in causal connectivity analysis.
