# Supplementary material for: Dynamic Construction of Stimulus Values in the Ventromedial Prefrontal Cortex
Source: PLoS One. 2011 Jun 14;6(6):e21074. doi: 10.1371/journal.pone.0021074 (PMC3114863; doi:10.1371/journal.pone.0021074)
Supplement: Table S2 — Peak MNI coordinates, source reconstruction 400–550 ms. Clusters surviving FWE-corrected threshold p<0.05 (F = 56.5) and cluster size threshold k = 5. * Denotes clusters used as ROIs in causal connectivity analysis. (DOC) [file pone.0021074.s007.doc]

**Table S2.** Peak MNI Coordinates, Source Reconstruction 400-550 ms.

| *# Voxels* | *Side* | *Peak MNI Coordinates* | | | *F* | *MNI Coordinate Region* |
| --- | --- | --- | --- | --- | --- | --- |
| 716 | L | **–6** | **14** | **–20** | **215.6** | Medial frontal gyrus* |
|  |  | –4 | 4 | –6 | 185.6 | Rectal gyrus |
|  |  | –32 | 2 | –16 | 177.1 | Ventral striatum |
| 364 | R | **10** | **22** | **–18** | **205.4** | Medial frontal gyrus* |
|  |  | 12 | 2 | –14 | 190.8 | Rectal gyrus |
|  |  | 14 | –6 | –14 | 182.7 | Ventral striatum |
| 12 | R | **56** | **–20** | **–14** | **90.0** | Middle temporal gyrus |
| 6 | L | **–8** | **–10** | **30** | **78.4** | Cingulate gyrus |
| 8 | R | **54** | **6** | **–28** | **72.4** | Anterior temporal lobe |
| 26 | R | **42** | **14** | **–30** | **71.8** | Anterior temporal lobe |
| 9 | L | **–56** | **–12** | **–24** | **71.4** | Inferior temporal gyrus |
| 18 | L | **–30** | **–48** | **–6** | **71.2** | Parahippocampal gyrus |
| 18 | L | **–38** | **44** | **18** | **64.8** | Dorsolateral prefrontal cortex* |
|  |  |  |  |  |  | BA46 |
| 5 | L | **–36** | **2** | **–24** | **59.8** | Medial temporal cortex |
| 5 | R | **8** | **–80** | **44** | **57.4** | Precuneus |
| 5 | R | **10** | **–34** | **0** | **56.5** | Hippocampus |

Clusters surviving FWE-corrected threshold *p* < 0.05 (*F* = 56.5) and cluster size threshold *k* = 5. * Denotes clusters used as ROIs in causal connectivity analysis.
