# Supplementary material for: Dynamic Construction of Stimulus Values in the Ventromedial Prefrontal Cortex
Source: PLoS One. 2011 Jun 14;6(6):e21074. doi: 10.1371/journal.pone.0021074 (PMC3114863; doi:10.1371/journal.pone.0021074)
Supplement: Table S3 — Peak MNI coordinates, source reconstruction 700–800 ms. Clusters surviving FWE-corrected threshold p<0.05 (F = 55.9) and cluster size threshold k = 5. * Denotes clusters used as ROIs in causal connectivity analysis. (DOC) [file pone.0021074.s008.doc]

**Table S3.** Peak MNI Coordinates, Source Reconstruction 700-800 ms.

| *# Voxels* | *Side* | *Peak MNI Coordinates* | | | *F* | *MNI Coordinate Region* |
| --- | --- | --- | --- | --- | --- | --- |
| 257 | R | **6** | **26** | **–28** | **197.9** | Rectal gyrus |
|  |  | 32 | 8 | –16 | 94.2 | Medial frontal gyrus |
|  |  | 14 | 20 | –20 | 89.1 | Inferior frontal gyrus |
| 105 | L | **–8** | **28** | **–22** | **163.8** | Rectal gyrus |
|  |  | –4 | 4 | –6 | 79.0 | Medial frontal gyrus |
|  |  | –6 | 14 | –14 | 77.8 | Ventral striatum |
| 49 | R | **38** | **48** | **6** | **116.6** | Middle frontal gyrus |
|  |  | 38 | 46 | 14 | 64.6 | Dorsolateral prefrontal cortex |
| 66 | R | **44** | **16** | **–24** | **102.5** | Anterior temporal lobe |
|  |  | 40 | 0 | –18 | 92.4 |  |
| 7 | L | **–36** | **–8** | **2** | **93.4** | Insula |
| 15 | R | **22** | **4** | **50** | **80.5** | Frontal lobe |
| 21 | L | **–54** | **–12** | **–22** | **79.0** | Middle temporal gyrus |
| 26 | R | **24** | **–64** | **50** | **78.3** | *Intraparietal sulcus |
| 6 | L | **–36** | **–6** | **8** | **77.8** | Insula |
| 40 | L | **–26** | **14** | **–14** | **73.0** | Inferior frontal gyrus |
|  |  | –28 | 14 | –24 | 62.0 | Insula |
| 26 | R | **40** | **40** | **28** | **72.3** | Middle frontal gyrus |
|  |  | 34 | 46 | 32 | 67.3 |  |
| 5 | R | **36** | **–16** | **4** | **69.8** | Insula |
| 12 | L | **–22** | **52** | **0** | **69.3** | Superior frontal gyrus |
| 15 | R | **54** | **22** | **16** | **67.7** | Inferior frontal gyrus |
| 10 | R | **48** | **22** | **8** | **64.2** | Inferior frontal gyrus |
| 5 | R | **54** | **–18** | **–18** | **63.3** | Middle temporal gyrus |
| 7 | L | **–32** | **–2** | **–18** | **63.1** | Amygdala |

Clusters surviving FWE-corrected threshold *p* < 0.05 (*F* = 55.9) and cluster size threshold *k* = 5. * Denotes clusters used as ROIs in causal connectivity analysis.
